# Real-time ultrasound-guided stellate ganglion block for migraine: an observational study

**DOI:** 10.1186/s12871-022-01622-8

**Published:** 2022-03-24

**Authors:** Jiawei Hou, Shaofeng Pu, Xingguo Xu, Zhiqiang Lu, Junzhen Wu

**Affiliations:** 1 Department of Anesthesiology, The Third People’s Hospital of Tongzhou District, Nantong City, 226311 Jiangsu Province China; 2 Department of Pain Management, Shanghai Jiao Tong University Affiliated Sixth People’s Hospital, Shanghai, 200233 China; 3 Department of Anesthesiology, Affiliated Hospital of Nantong University, Nantong, 226001 China; 4 Department of Anesthesiology, Shanghai Eighth People’s Hospital, Shanghai, 200233 China

**Keywords:** Ultrasound guidance, Stellate ganglion block (SGB), Migraine

## Abstract

**Objective:**

To observe whether ultrasound-guided stellate ganglion block (SGB) can effectively relieve migraine pain and improve the quality of migraine patients’ life.

**Methods:**

81 patients with migraines were enrolled in this study. The patients received SGB with 6 ml of 0.15% ropivacaine once every week for four times. Migraine was assessed with the Migraine Disability Assessment Scale (MIDAS) at baseline and three-months follow-up (Tm). The numerical rating scale (NRS) score at baseline, one day after treatment (Td) and Tm, the frequency of analgesic use in 3 months and the side effects were also recorded at the same time.

**Results:**

The NRS score of migraine subjects decreased significantly from 7.0 (2.0) to 3.0 (1.0) at Td and 2.0 (2.0) at Tm (vs baseline, *P* < 0.01). The MIDAS total scores were 14.0 (10.5) at baseline and 7.0 (4.5) at Tm (*P* < 0.001). During the three months, the frequency of analgesic consumption was decreased from 6.2 ± 2.8 to 1.9 ± 1.8. There were no serious side effects.

**Conclusions:**

This study confirmed that ultrasound-guided SGB is an effective method to treat migraines. This technique can reduce pain and disability and then improve the quality of life of patients with migraines.

## Introduction

Migraine is a nervous system disease characterized by headache, nausea, vomiting and sensitivity to visual, auditory, olfactory and skin irritation. It is usually recurrent with moderate to severe pain and poor drug control. Migraine is the third leading cause of disability globally. It affects hundreds of millions of people and accounts for about 10% of the world’s population [1, 2]. In addition, migraine-related disability and malpractice cause substantial financial costs [3]. Although there are some drugs for acute treatment and prevention of migraine, other treatment options for patients with poor drug response or intolerance to drug treatment should be considered [4].

Stellate ganglion block (SGB) is a safe procedure that may provide extended relief for breast cancer-related lymphedema (BCRL) and all clusters of Post-Traumatic Stress Disorder (PTSD) symptoms [5, 6]. SGB is also an accepted intervention for the treatment of various pain conditions of the head and neck regions as well as the upper limbs [7]. The stellate ganglion is a sympathetic ganglion, approximately 2.5 cm in length, 1 cm in width and 0.5 cm in thickness. It is located in front of the neck of the first rib and can extend to the seventh cervical spine (C7) [8]. Yu et al. reported that ultrasound-guided SGB could effectively relieve cervical headache [9]. However, the time point of pain assessment was 1 day after SGB, with no long-term follow-up data. A successful case of stellate ganglion block for difficult therapy of refractory tension headache was reported in 2019 [10]. One study showed that bilateral SGB was effective to reduce pain and improve the Migraine Disability Assessment Scale (MIDAS) score in two migraine patients [4]. However, Evidence regarding the effect of SGB on migraines is scanty. Herein, we investigate whether SGB can reduce acute migraine attacks and whether it can effectively reduce the frequency of migraine attacks within three months.

## Methods

Patients were enrolled between June 15, 2017 and March 20, 2019. The inclusion criteria were participants over 18 years old, diagnosed with migraine. Diagnoses of migraine were made by expert physicians according to the ICHD criteria [11].

The exclusion criteria included patients with a space-occupying lesion, coagulation disorders, systemic or local infection and drug allergies. Psychotic patients and migraine with bilateral attacks were also excluded from this study.

All patients were administered with SGB on the affected side and 0.15% ropivacaine was injected. SGB was conducted once a week for 4 times. All patients were followed up for 3 months. Analgesics such as diclofenac sodium were taken orally if the migraine attacks and the pain was serious, but no more than 10 times per month to avoid medication overuse headache.

### Data recording

The primary outcome was the MIDAS score at 3-months follow-up (Tm). The MIDAS questionnaire is one of the most widely used to measure the decline of quality of life caused by migraine [12]. The MIDAS questionnaire is a short, self-administered questionnaire designed to quantify headache-related disability within 3 months. It includes five questions about work, housework and non-work activity (social, family and leisure activities) to assess the degree of disability caused by headaches. The MIDAS score is closely related to the judgment of the severity of the headache and the need for medical care [13] and has been widely used in China [14–16].

Secondary outcomes include the numerical rating scale (NRS) score at baseline, one day after treatment (Td) and 3 months after treatment (Tm). The NRS allows the subject to rate their pain on an eleven-point numerical scale. The scale is composed of 0 (no pain at all) to 10 (worst imaginable pain) [17]. We defined migraine with an NRS score > 7 as severe migraine. The frequency of non-steroidal anti-inflammatory drugs (NSAIDs) consumption in 3 months was also recorded. During the follow-up period, SGB-related side effects such as hoarseness, dysphagia and foreign body sensation in the throat, upper limb weakness and hematoma formation were confirmed and recorded by doctors. Serious complications such as general spinal anesthesia, epidural block and pneumothorax were reported to the ethics committee. Patients were asked to inform doctors of any adverse symptoms they had experienced at any time.

### Procedures

#### Ultrasound-guided SGB

**Fig. 1:**
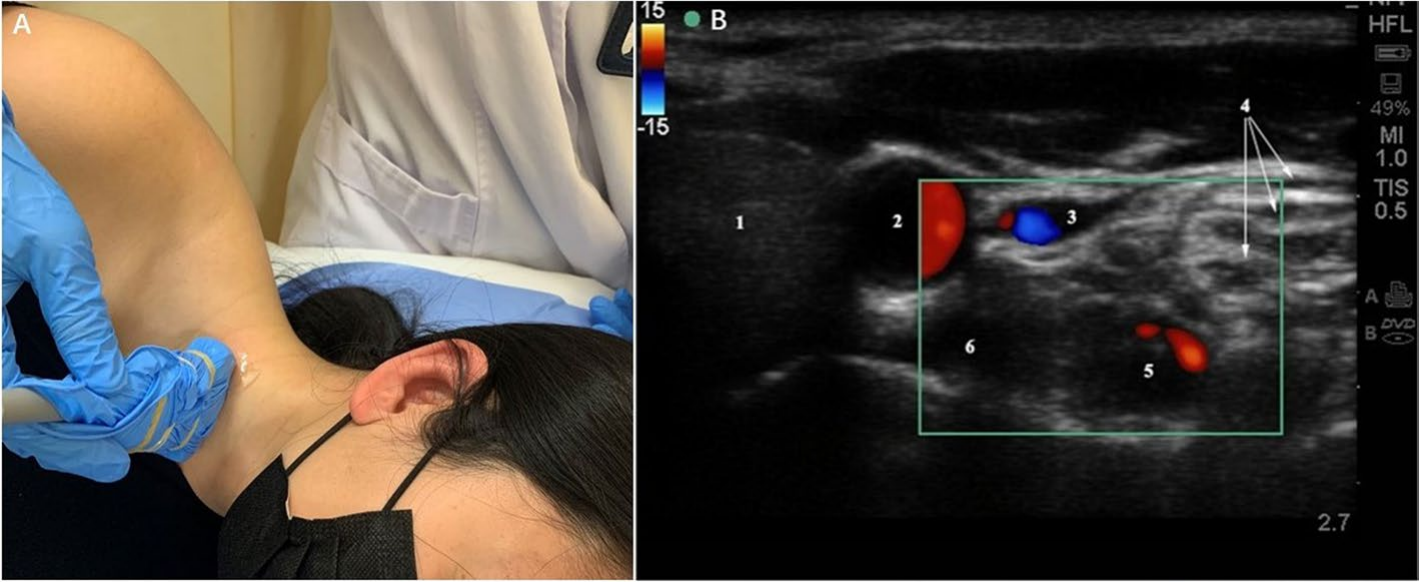
Ultrasonic exploration of stellate ganglion. **A**: Patient’s position and placement of ultrasound probe. **B**: Ultrasound image of the cervical structure during stellate ganglion block.1, thyroid; 2, common carotid artery; 3, internal jugular vein; 4, brachial plexus; 5, vertebral artery; 6, longus colli muscle

The patient’s position was similar to that of the traditional blind method. Patients were positioned in a lateral position with their necks slightly hyperextended. Assisted by ultrasound imaging equipment and a 7–14 MHz linear array probe (S-Nerve, SonoSite, USA), the C7 level was confirmed. Because of the tiny or absent anterior tubercle of the C7, sonoanatomy of the C7 transverse process was similar to the traditional Chinese “imperial concubine chair”. The thyroid gland, carotid artery, compressible internal jugular vein, vertebral artery, brachial plexus and the oval-shaped structure of the longus colli muscle were revealed on this short-axis view. The color Doppler mode was used before needling to avoid penetrating blood vessels such as the vertebral artery, internal jugular vein and inferior thyroid vessel (Fig. 1).

**Fig. 2:**
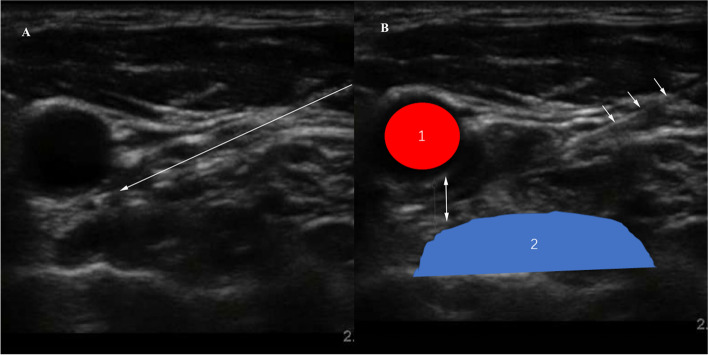
Stellate ganglion block process. **A**: The tip of the needle reached the surface of the longus colli muscle and the 5 o’clock position of the carotid artery. **B**: After administration of ropivacaine, the carotid artery can be seen floating like a balloon in real time under ultrasound, which indicates that the ropivacaine diffuses in the paravertebral space and can effectively block the stellate ganglion. 1, common carotid artery; 2, longus colli muscle; The three arrows indicate the puncture needle, and the two-way arrows indicate that the distance between the common carotid artery and the longus colli muscle is larger than that in Fig. **A**

Ultrasound can provide the perfect technical means to achieve this goal. We used a 25-gauge, 8-cm needle for a puncture, and the puncture point was 1–1.5 cm away from the ultrasound probe. The in-plane puncture technique was used for real-time display of the whole process of puncture. The tip of the needle reached the surface of the longus colli muscle and the 5 o’clock position of the carotid artery (Fig. 2a). Under the guidance of the ultrasound, SGB was performed by injection of 6 ml of 1.5% ropivacaine. The common carotid artery was observed “floating” upward under ultrasound, indicating that the drug diffused in the prevertebral space on the surface of the longus colli muscle (Fig. 2b). All measurements were performed by a senior anesthesiologist using the same ultrasound instrument.

### Statistical analysis

MIDAS and NRS scores are expressed as median with interquartile range (IQR). A generalized linear mixed model (GLMM) was performed to evaluate changes in pain NRS scores over repeated measurements. If the repeated measures demonstrated a statistically significant time interaction, multiple comparison corrections were performed using Bonferroni correction. Changes in migraine outcomes as assessed by the MIDAS questionnaire were compared using a paired Wilcoxon test or signed rank-sum test. A *P*-value < 0.05 was considered statistically significant. Statistical analyses were conducted using SPSS 18.0 software for Windows (SPSS Inc., Chicago, IL, USA).

## Results

### Subject characteristics

**Fig. 3:**
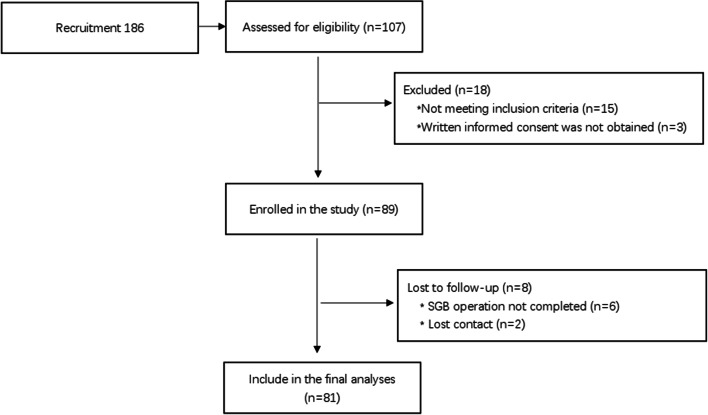
Flow chart for selecting study population

We recruit 186 subjects. After preliminary investigation, 107 subjects were assessed for eligibility. However, among 107 subjects, 15 subjects did not meet the inclusion criteria and the informed consent form was not obtained from 5 subjects. Therefore, 86 subjects were finally enrolled in the study. Of 86 patients, 6 patients did not complete all the four SGB and 2 patients lost follow-up, and therefore 81 patients (23 men, 58 women; median age, 33.2 ± 7.9 years; range, 18–62 years) were included in the final analysis (Fig. 3).

Within three months, 17 patients had severe migraine attacks < 3 times, 44 patients had severe migraine attacks 3–6 times, 11 patients had severe migraine attacks 7–12 times and 9 patients had severe migraine attacks > 12 times (Table 1).

**Table 1:** Demographic characteristics

	Frequency (n)	Proportion (%)
Gender		
Male	58	71.6
Female	23	28.4
Age		
18–28	19	23.5
29–38	46	56.8
39–48	12	14.8
>48	4	4.9
Frequency of severe headache pre quarter (NRS>7)		
<3	17	21.0
3–6	44	54.3
7–12	11	13.6
>12	9	11.1

### Clinical assessment

MIDAS total scores of subjects were 14.0 (10.5) at baseline and 7.0 (4.5) at Tm (*P* < 0.001) (Table 2). NRS scores at baseline, Td and Tm were 7.0 (2.0), 3.0 (1.0) (vs baseline, *P* < 0.01) and 2.0 (2.0) (vs baseline, *P* < 0.01), respectively (Table 3). The frequency of analgesic use within 3 months was 6.2 ± 2.8 at baseline and 1.9 ± 1.8 at Tm.

**Table 2:** Migraine outcomes as assessed by the MIDAS questionnaire

MIDAS scores	SGB (*n* = 81)			
	Tb	Tm	Z-Value	*p*-Value
Total score	14.0 (10.5)	7.0 (4.5)	7.553	< 0.001
On how many days in the last 3 months did you miss work or school because of your headaches?	1.0 (1.0)	0.0 (0.0)	5.781	< 0.001
How many days in the last 3 months was your productivity at work or school reduced by half or more because of your headaches?	3.0 (2.0)	3.0 (2.0)	5.955	< 0.001
On how many days in the last 3 months did you not do household work because of your headaches?	3.0 (3.0)	1.0 (2.0)	7.026	< 0.001
How many days in the last 3 months was your productivity in household work reduced by half or more because of your headaches?	6.0 (5.0)	2.0 (2.0)	7.656	< 0.001
On how many days in the last 3 months did you miss family, social, or leisure activities because of your headaches?	2.0 (2.0)	1.0 (2.0)	5.276	< 0.001

MIDAS Scores are expressed as median (IQR)

*Tb* Baseline, *Tm* Three-months follow-up, *MIDAS* Migraine Disability Assessment Scale, *SGB* stellate ganglion block

**Table 3:** NRS of migraine

	Tb (Baseline)	Td (24 h after treatment)	Tm (3 months follow-up)
NRS	7.0 (2.0)	3.0 (1.0)^*^	2.0 (2.0)^*^

Values present as median (IQR); ^*^p < 0.01, vs NRS at baseline; NRS, numeric pain scale

Of the 86 patients recruited, two patients developed headaches after receiving a single SGB treatment and gave up follow-up treatment. Among the 81 patients who were included in the final analysis, there were 6 cases of hoarseness, 3 cases of local bruise and 2 cases of transient upper limb numbness. There were no serious complications such as gastrorrhagia, general spinal anesthesia, epidural block and pneumothorax.

## Discussion

Although migraine is not the most frequent primary headache in the world, its incidence is quite high, affecting more than 10% of the world’s population. Migraine is usually unilateral and moderate to severe. It worsens with daily physical activities, such as walking and climbing stairs, seriously affecting patients’ work and quality of life [1].

In addition to prophylactic (e.g., flunarizine, topiramate and amitriptyline) and acute (e.g., diclofenac sodium and triptans) migraine therapies, treatment of migraine triggers and other lifestyle factors that may aggravate the migraine tendency in patients is critical. Specific behavioral therapies, including biofeedback, teaching relaxation techniques and cognitive behavioral therapy (CBT), are also beneficial [18]. However, these treatment modalities require patient education and, in some cases, specific behavioral skills. This may cause great confusion for elderly patients or patients who do not receive proper education.

Furthermore, the effect of nerve block on migraine has been documented. The cranial nerve block was an effective adjuvant therapy, which could reduce the intensity, duration and frequency of pain, and improve the satisfaction of migraine and central sensitization patients [19]. A large retrospective cohort study showed that greater occipital nerve block can effectively reduce migraine [20]. Li et al. reported a case of long-term effective treatment with pulsed radiofrequency of C2 dorsal root ganglion under ultrasound guidance [21]. The application of trigger point therapy in migraine has also been reported [22]. Moreover, SGB block has been confirmed to effectively treat migraine [4]. However, studies evaluating SGB in the treatment of migraine are limited, and even a case report shows that SGB causes migraine attacks [23]. In the present study, we found that ultrasound-guided SGB, once a week for four weeks, can significantly relieve the pain of migraine patients.

Ultrasound is a valuable tool for imaging soft tissue structures and nerves, guiding needle advancement and confirming the spread of the injectate around the target without exposing physicians and patients to the risks of radiation [24, 25]. There is a rapidly growing interest in ultrasound-guided SGB, as evidenced by the surging number of publications in the last few years. It has been reported that 2 ml of 2% mepivacaine can be effective when SGB is performed at the level of the 6th cervical transverse process under ultrasound [26]. About 6 ml of 0.15% ropivacaine was found to be sufficient for a successful SGB with fewer complications, which coincides with the present study [27].

The stellate ganglion is extensively connected with the cerebral cortex, hypothalamus, amygdala and hippocampus [28]. SGB can effectively treat postherpetic neuralgia, hot flushes and night awakenings in survivors of breast cancer, tension headache and PTSD, in part by improving blood supply and inhibiting the connection between the stellate ganglion and the brain through sympathetic action within its innervation; however, the exact mechanism remains unclear [5, 6, 10, 29–31]. Low melatonin levels have been reported in migraine patients and SGB has been shown to restore melatonin rhythm. Melatonin can effectively prevent migraines by inhibiting the synthesis of nitric oxide and the release of calcitonin-related peptides and antagonizing excitotoxicity caused by glutamate [32–34]. In addition, stress is the most common migraine trigger. In response to stress, sympathetic activity increases, leading to the release of migraine-associated neurotransmitters, such as dopamine and prostaglandins [35–37]. High dopamine levels can lead to nausea and vomiting, while increased prostaglandins can increase pain sensitivity and inflammation in migraine patients. SGB can regulate sympathetic nerve activity, thereby alleviating most of the symptoms in migraine patients. In this study, we did not detect melatonin, prostaglandins and dopamine levels as in previous studies. We look forward to further research in this field in the future.

MIDAS was designed to quantify headache-related disability over 3 months. The reliability and internal consistency of the MIDAS score are comparable to those of a previous questionnaire (Headache Impact Questionnaire). However, the MIDAS score requires fewer questions, is easier to score and provides intuitively meaningful information on lost days of activity in three domains [38, 39]. The MIDAS questionnaire was considered highly reliable and effective and was relevant to clinical judgment on medical care needs. In our study, the MIDAS score of patients was significantly decreased at 3-months follow-up. Besides, NRS scores decreased significantly one day and 3 months after SGB. The frequency of analgesic use was also significantly decreased after 3 months. These results suggest that SGB once a week for 4 weeks can reduce the headache and disability among migraine patients and improve their work and quality of life.

Two patients who were recruited but not included in the analysis experienced migraine aggravation after a single SGB treatment, suggesting that migraine has more complex mechanisms. Wulf et al. detailed complications after SGB. Most of them were related to the central nervous system (such as convulsions). Other serious complications included high-level subarachnoid block, high-level epidural block, pneumothorax and allergic reaction. All SGBs were performed without fluoroscopy [40]. In our study, serious complications such as subarachnoid block, epidural block and convulsion were not observed. It is suggested that ultrasound-guided SGB is safer than fluoroscopy because ultrasound can clearly distinguish nerves, blood vessels and muscles, and monitor the puncture needle in real-time, while fluoroscopy has no such functions.

## Conclusions

In summary, the findings of this study suggest that real-time ultrasound-guided SGB may be an effective treatment option for migraine patients without serious complications. However, further studies should be performed to verify this hypothesis.

### Limitations

This study was not a randomized controlled study, and only a few cases were included.

## Data Availability

We are willing to share individual deidentified participant data, such as the age of the subjects and MAIDS score and NRS score. We will share the data within three months after the accept of the article and keep it for at least three years. The data can be accessible at www.medresman.org.cn/pub/cn/proj/searchSH.aspx.
